# THE IMPACT OF FAR-INFRARED TECHNOLOGY ON QUALITY OF LIFE IN OLDER ADULTS

**DOI:** 10.1093/geroni/igad104.3133

**Published:** 2023-12-21

**Authors:** Shavonnye Rath, Joshua Guggenheimer, Melanie Homan, Marcella Myers, Jennifer Hutson, Ginny Green

**Affiliations:** St. Catherine University, St. Paul, Minnesota, United States; St. Catherine University, St. Paul, Minnesota, United States; St. Catherine University, St. Paul, Minnesota, United States; St. Catherine University, St. Paul, Minnesota, United States; St. Catherine University, St. Paul, Minnesota, United States; St. Catherine University, St. Paul, Minnesota, United States

## Abstract

Identifying methods of pain amelioration to meet the unique demands of older adults (OA) may be crucial to increasing quality of life (QOL) for this population. This study aimed to examine the effects of far-infrared heat (FIR) on pain management and QOL in OA. FIR utilizes a long light wavelength that simulates dry sauna-like conditions. Examining the relationship between FIR and pain is important due to the increased prevalence of chronic pain associated with aging and the corresponding impact it has on QOL and physical performance. Seventeen OA completed the study. Participants were randomly assigned to either a convective heat group (CON) or a convective and far-infrared heat (FIR) group, with convective heat set to 60℃ (140℉.) Participants received six, 30-minute sessions. Physical assessments were measured (range of motion, gait speed, timed up-and-go, and hand grip strength) as well as pain severity and its interference with daily life, and the impact pain had on overall QOL. T-tests were used to compare pre- and post-assessment measures. Results indicated that pain severity was significantly reduced (from 3.31 to 2.5, P < 0.05) in the FIR group and that pain interference was significantly reduced (from 1.26 to 0.43, P < 0.05) in the CON group. Findings suggest that heat therapy successfully reduced pain over time, but that FIR heat specifically was not superior to that of convective heat alone. Further research is required to correctly identify the relationship between QOL and the use of FIR.

